# Dynamics of capillary infiltration of liquids into a highly aligned multi-walled carbon nanotube film

**DOI:** 10.3762/bjnano.2.36

**Published:** 2011-06-20

**Authors:** Sławomir Boncel, Krzysztof Z Walczak, Krzysztof K K Koziol

**Affiliations:** 1 Department of Organic Chemistry, Bioorganic Chemistry and Biotechnology, Silesian University of Technology, Krzywoustego 4, Gliwice 44-100, Poland; 2 University of Cambridge, Department of Materials Science and Metallurgy, Pembroke Street, Cambridge CB2 3QZ, United Kingdom

**Keywords:** capillary action, dynamic viscosity, highly aligned carbon nanotubes, superhydrophobicity, wettability

## Abstract

The physical compatibility of a highly aligned carbon nanotube (HACNT) film with liquids was established using a fast and convenient experimental protocol. Two parameters were found to be decisive for the infiltration process. For a given density of nanotube packing, the thermodynamics of the infiltration process (wettability) were described by the contact angle between the nanotube wall and a liquid meniscus (θ). Once the wettability criterion (θ < 90°) was met, the HACNT film (of free volume equal to 91%) was penetrated gradually by the liquid in a rate that can be linearly correlated to dynamic viscosity of the liquid (η). The experimental results follow the classical theory of capillarity for a steady process (Lucas–Washburn law), where the nanoscale capillary force, here supported by gravity, is compensated by viscous drag. This most general theory of capillarity can be applied in a prediction of both wettability of HACNT films and the dynamics of capillary rise in the intertube space in various technological applications.

## Introduction

Wettability of carbon nanotubes (CNTs) and highly aligned carbon nanotube (HACNT) films is an important aspect in numerous technologies including manufacture of composites [1], fabrication of constantly/interchangeably hydrophobic or hydrophilic materials [2–3], nanofluidic devices [4] or sponges for non-polar liquids [5]. One of the cutting edge areas of research exploiting CNTs is nanomedicine where the interface of CNTs with a liquid environment is essential, e.g., subcutaneous glucose sensors [6], microcatheters [7] or tissue engineering materials [8]. Until now, physical compatibility of liquids and pristine CNTs was determined by dispersibility of randomly oriented and highly entangled, hydrophobic nanotubes. Wetting of CNTs in non-polar to medium polar liquids (a key factor enabling their dispersibility) can be gained generally via two routes: (1) By control of the CNTs dimensions, either in the stage of synthesis [9], or in a stage of separation of nanotube bundles, e.g., in a prolonged and vigorous ultrasonication (with frequent nanotube cutting as an accompanying process) [10], or (2) via chemical modification of the nanotube surface [11]. A simple chemical treatment of CNTs can entirely alter their interface performance, from highly lipophilic to even lipophobic [12–13]. In turn, preparation of aqueous dispersions of CNTs requires the use of low or high molecular weight surfactants [14].

Most of the theoretical approaches concerning dispersibility of CNTs in various liquids specify the Hildebrand solubility parameter (δ) as crucial towards convenient three-modal classification: dispersion, swelling or precipitation [15–18]. For evaluation of wettability of HACNT arrays, an approach based on the direct measurement of contact angle between interfaces of nanotube film and liquids was applied [19–21]. In order to verify critical parameters for wetting of HACNT films, we present here a simple and clear-cut approach. Our concept is based on a combination of thermodynamic and kinetic stipulation. The first parameter, the contact angle between HACNT film and the meniscus of a given liquid, defines eventuality of infiltration. The second parameter, dynamic viscosity of the liquid, corresponds to the rate of infiltration or, in other words, dynamics of the capillary rise in the intertube space. As a model of HACNT film we used a CVD-grown array of a specified nanotube density and of a confirmed degree of alignment. The film was subsequently treated with droplets of various liquids, under carefully selected conditions. The output of the experiment, that is, time of infiltration as a function of the applied liquid, was analysed using the most general theoretical model, namely the Lucas–Washburn law.

## Results and Discussion

**Figure 1:**
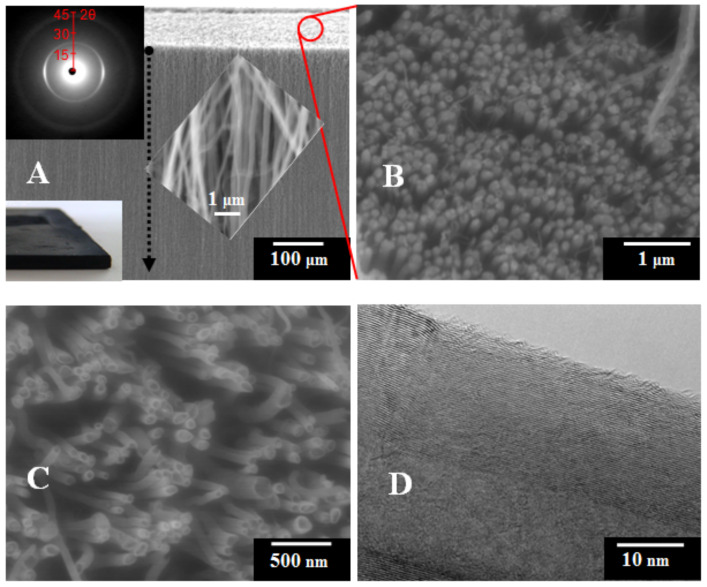
SEM micrographs (A–C): A side (A) and a top view (B) of the HACNT film grown on a quartz substrate; (C) a magnified top view of the film shows nanotube tips closed with iron catalyst nanoparticles, suggesting that CNTs were grown according both to tip-to-base and base-to-tip growth mechanisms; a representative TEM image (D) of a nanotube within the HACNT array. Insets in (A): lower left – a photograph of the as-grown HACNT array; upper left corner – a 2D wide-angle X-ray diffractogram of the HACNT film; central part – a side-view magnification showing degree of alignment. A dashed arrow indicates the flow direction of tested liquids, opposite to the growth direction of the HACNT array.

With the aim of understanding the wetting behaviour of the HACNT film (5 × 10^6^ nanotubes/mm^2^ dense, corresponding to an intertube separation of ~440 nm and free volume of 91%) (Figure 1), a series of experiments was performed in which the film was put in contact with a variety of liquids. SEM images of the as-synthesised HACNT film (Figure 1A–1C) showed a high degree of alignment of nanotubes. The thickness of the film was equal to 0.74 ± 0.04 mm. Additionally, the alignment of the nanotubes was independently confirmed in a wide-angle X-ray diffraction (XRD) analysis, using the (002) reflection (at 2θ = 25.7°) corresponding to the graphene interlayer spacing of MWCNTs (inset in Figure 1A). The level of misalignment of nanotubes in the arrays was ±16° based on the azimuthal integration of the (002) reflection, indicating high alignment quality. It is worth noting that the spread convolutes variations in graphitic quality at the atomic scale, with overall variations in CNT orientation, and more complex diffraction effects are observed as disorder increases [22]. The mean outer and inner diameters of CNTs as measured by TEM were 60 ± 25 and 10 ± 2 nm, respectively. Finally, a representative TEM image revealing multi-walled structure of a nanotube with characteristic interstices between adjacent graphene layers of 0.34 nm is shown in Figure 1D.

In the key experiments, a single drop (10 µL) of each liquid was dosed using a micropipette from ~0.5 cm height onto the surface of the pure HACNT film, supported, as-grown, on a quartz substrate. The drops were always dosed at fresh locations onto the film. For each liquid the infiltration time (*t*) was measured three times. The measurements were based simply on the disappearance of particular droplets from the surface of the HACNT film. The results of infiltration are presented in Table 1.

**Table 1:** Infiltration time (*t*) of the HACNT film with different liquids as a result of the combination of a thermodynamic prerequisite (the contact angle, θ) and a kinetic stipulation (the viscosity, η). γ_L_ is the surface tension, and δ is the Hildebrand solubility parameter.

**Material**	**δ [MPa^1/2^] **[17]				

CNTs	20.8				

**Liquid**	**δ [MPa^1/2^] **[23]	**γ_L_ [mN∙m^−1^] **[24]	**η [Pa∙s] **[25]	**θ [°]**	***t* [s]**

diethyl ether	15.1	16.7	0.00024	<90	<1
acetonitrile	24.3	28.7	0.00036	<90	<1
dichloromethane	20.2	27.8	0.00043	<90	<1
ethyl acetate	18.1	23.2	0.00046	<90	<1
methanol	29.6	22.1	0.00054	<90	<1
chloroform	18.7	26.7	0.00056	<90	<1
*N*,*N*-dimethylformamide (DMF)	24.8	34.4	0.00080	<90	<1
styrene	19.0	32.0	0.00081	<90	<1
1 wt % SDBS^a^ (aq)	n/a	33.8^b^	0.0010	<90	<1
*n*-decane	16.0	23.8	0.00168	<90	<1
formic acid	24.9	37.7	0.0018	<90	<1
*N*-methyl-2-pyrrolidone (NMP)	22.9	44.6	0.0018	<90	<1
dimethyl sulfoxide (DMSO)	26.7	42.9	0.00200	<90	<1
ethylene glycol	34.9	42.9	0.026	<90	18
PEG 400^c^	17.6	58.5	0.050	<90	54
paraffin oil^d^	16.5	26.0	0.100	<90	150
saturated sucrose (aq)	n/a	76.5	0.3900	<90	240
silicon oil^e^	9.7	21.5	0.625	<90	960
glycerine	36.2	76.2	1.070	<90	1080
water	48.0	72.7	0.0010	165 ± 5	+∞
saturated sodium chloride (aq)	>48.0	82.5	0.0029	165 ± 5	+∞
mercury	63.0	474.4	0.00152	175 ± 5	+∞

^a^An industrial mixture of isomers with the formula C_12_H_25_C_6_H_4_SO_3_^−^Na^+^; ^b^cf. [26]; ^c^polyethylene glycol, a low molecular weight grade of polyethylene glycol of formula H–(CH_2_CH_2_)*_n_*–OH, *n* = 8–9; ^d^C_17_–C_30_, CAS 8042-47-5; ^e^ρ = 1.05 g/cm^3^, (H_3_C)[Si(CH_3_)_2_O]*_n_*Si(CH_3_)_3_.

**Figure 2:**
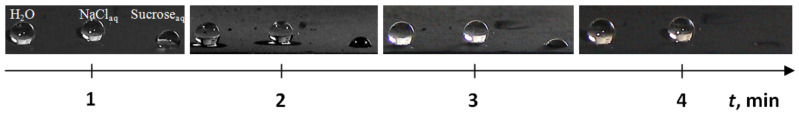
Sequence of images showing infiltration of the HACNT film, as grown on the quartz substrate, by different aqueous solutions. Each image contains a droplet of water (left), saturated saline solution (centre) and saturated sucrose aqueous solution (right).

All of the examined liquids, except for water, saturated saline (NaCl) and mercury, infiltrated the nanotube film. A comparative sequence of snapshots is presented in Figure 2 showing the infiltration of the HACNT film by three different aqueous solutions, characterised by various dielectric constant, namely distilled water (as a reference) (left), saturated saline (centre) and saturated sucrose solution (right).

Water and saline solution both formed droplets that slide on the highly hydrophobic HACNT array. The droplets could be fully removed from the CNT surface using a paper tissue. The contact angle (θ) between the HACNT film and water (as well as saline) in air, as measured from magnified video shots, was constant over a period of 5 minutes and was equal to 165 ± 5°. This value allows us to qualify the herein considered HACNTs film as a “superhydrophobic” material [27]. In contrast, an unexpected behaviour in the series of aqueous solutions was observed for sucrose, which slowly, but entirely, infiltrated the HACNT film.

Theoretically, two requirements for a successful infiltration of HACNT array by a liquid can be identified. Firstly, the process must be thermodynamically favourable. For a given nanotube density within a HACNT film, which is a material characterised by a large capillarity [28], it is critical that the liquid wets the CNT surface [29–30]. In general, the dispersibility of nanoparticles is achieved when the dispersion components do not differ considerably (typically less than ~5 MPa^1/2^) in the Hildebrand solubility parameter (δ, MPa^1/2^): δ^2^ = δ_d_^2^ + δ_p_^2^ + δ_h_^2^, where δ_d_, δ_p_ and δ_h_ represent the dispersive, polar and hydrogen bonding interaction components (Hansen solubility parameters), respectively [31]. The Hildebrand solubility parameter for single-walled carbon nanotubes was estimated by plotting the nanotube dispersibility as a function of the Hansen solubility parameters for the solvents that were capable of infiltration. These solvents occupied a well-defined range of Hansen parameter space. Although the authors estimated the dispersion, polar, and hydrogen bonding Hansen parameter for the nanotubes to be δ_d_ = 17.8 MPa^1/2^, δ_p_ = 7.5 MPa^1/2^, and δ_h_ = 7.6 MPa^1/2^, the nature of the distinction between solvents and non-solvents of CNTs remains to be fully understood [17]. The Hildebrand solubility parameter, expressed as a square root of cohesive energy density, can also be correlated with the surface tension (γ_L_) [32], e.g., by the Equation 1 (where *V*_L_ is molar volume of a liquid) [33–34]:

[1]
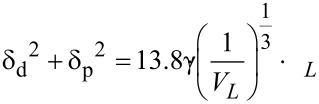


However, a critical parameter for the flow of a liquid through capillary action, found from the Newton dynamic equation, is the contact angle (θ). The contact angle is simply a measure of the difference in affinities for liquid–liquid and solid–liquid phases. Once the liquid wets the solid phase, it will rise to a stationary level in the capillary channel (*z*_∞_) established by the balance between gravitational and capillarity force:

[2]
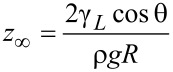


where *R* is the average pore size.

The second factor affecting the infiltration, via the kinetics of this rise (the rate of infiltration), is expected to be the viscosity of the liquid (at the infiltration temperature). The Lucas–Washburn law refers to a quasi-steady state of the liquid flow by the capillary action, where capillary force, expressed by the above thermodynamic parameters, contact angle (θ) and surface tension (γ_L_), is compensated by gravity and viscous drag [35]. The height of the meniscus of the infiltrating liquid inside the capillary is a function of time (*z*(*t*)) according to Equation 3.

[3]
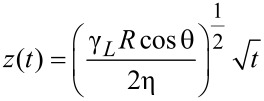


**Figure 3:**
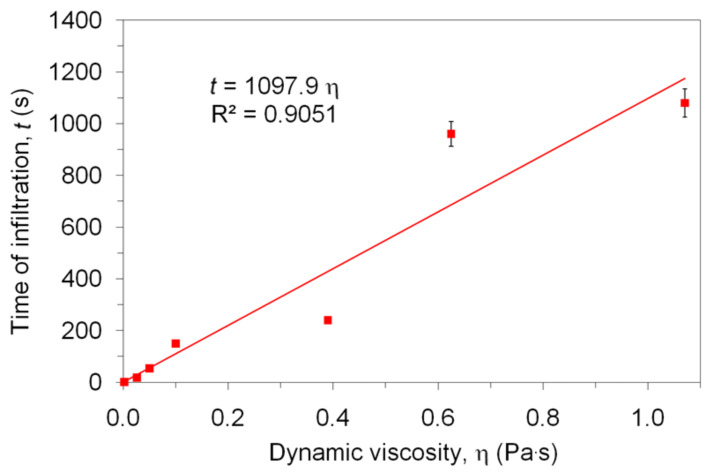
Time of infiltration of HACNTs film as a function of the dynamic viscosity of a range of viscous liquids.

The Lucas–Washburn law was found to hold also for capillary rise in nanopores [36]. This allows us, therefore, to correlate the time of infiltration (*t*) of the HACNT film with the dynamic viscosity (η) of liquids over a range of polarities (represented by Hildebrand solubility parameter, δ) (Table 1). As seen, the infiltration by organic liquids with a low viscosity, in the range from 0.00024 to 0.002 Pa·s, was practically instantaneous (described as <1 second). In the case of organic liquids with an order of magnitude higher viscosity (>0.026 Pa·s) the timescale was minutes. In general, and in agreement with intuitive presumptions, the higher the viscosity of the organic liquid, the slower the infiltration rate. In all of the above cases, the thermodynamic criterion (θ < 90°) was fulfilled and an empirical relationship between the rate of infiltration and dynamic viscosity (in the viscosity range from 0.026 to 1.07 Pa·s) was found based on the linear proportionality expected from the Lucas–Washburn equation (Figure 3).

The slope in the linear regression (1097.9 Pa^−1^) is defined by a combination of both invariable (penetration depth, radius of the channel) and variable parameters (surface tension and contact angle) (Equation 4):

[4]
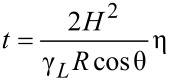


were *H* is the final height of the liquid in the capillary. The values of the surface tension for all infiltrating liquids are all within the same order of magnitude. The values of contact angle for these liquids, according to the theoretical background, must be lower than 90°. The *H*^2^/*R* ratio, appearing in the coefficient of proportionality in Equation 4, must be treated carefully as coalescence of nanotubes was observed. The coalescence manifested by a slight depression of the nanotube film at the point of infiltration of the liquid. Moreover, it is known that wetting of the nanotube walls by non-polar liquids, e.g., acetone, can increase the nanotube density [37]. This phenomenon was observed when the HACNT film was removed from the silica substrate and subjected to an analogous infiltration experiment, after which the film was bent into an oval curvature. Liquids do not enter the central internal cavity within the nanotubes produced by CVD synthesis, which are typically capped structures. Therefore, the only volume available for penetration by the liquids is the intertube space. The intertube separation distance calculated from the nanotube density in the HACNT film is ~310 nm, whereas an average pore size (*R*) derived from the *H*^2^/*R* ratio (20 m) is ~50 nm. This is also a reflection of the coalescence behaviour.

Addition of a conventional anionic surfactant, sodium dodecylbenzene sulfate (SDBS), to water (1% by weight), ensures good wetting of the nanotubes allowing the infiltration to occur. Saturated sucrose solution infiltrates the HACNT arrays but in considerably prolonged time. This behaviour can be explained if sucrose is considered as a non-ionic surfactant forming a 3D-network, via hydrogen bonds, entangling individual CNTs. It was found recently that polysaccharides, wrapped around nanotubes, enabled their “solubility” to various levels of unbundling (rope-to-single-tube transition) [38]. The best dispersions were obtained when gum arabic was used as a natural surfactant [39]. In order to draw further conclusions for the water-based systems a variety of surfactants with different concentrations would have to be examined.

## Conclusion

Infiltration by a variety of organic liquids, aqueous ionic/non-ionic solutions and metallic mercury into a model HACNT film was studied. In the case of organic liquids, which range from non-polar (9.7 MPa^1/2^) to highly polar (34.9 MPa^1/2^), wetting the nanotube walls, infiltration always took place. An empirical relationship between the rate of infiltration and viscosity of the liquid was found basing on the Lucas–Washburn equation. The elaborated equation is applicable in more complex systems, e.g., for an initial selection of appropriate polymer matrix, as a melt or a solution, and further processing towards aligned nanotube composites [40]. Since neither pure water, nor aqueous ionic solutions penetrate the HACNT film, this material can potentially be used in a production of ultra-light hydrophobic composites exhibiting other superb physicochemical properties originating from the as-synthesised nanotubes.

## Experimental

A solution of ferrocene (6 wt %, 98%, Acros Organics) in toluene (spectrophotometric grade, *d* = 0.86 g/cm^3^, Acros Organics) was injected into an argon atmosphere at 760 °C. CNTs grew both on the quartz reactor tube and on flat quartz substrates in a CVD furnace over 4 h. After the synthesis, the quartz reactor was cooled down in argon atmosphere. The HACNTs array grown on the flat quartz substrate was used without further treatment.

The nanotube density in the HACNT film was measured using ImageJ^®^ on an SEM image (sample with 100 nanotubes). The average diameters of MWCNTs were measured on TEM images of 100 nanotubes. Free volume of the HACNT film was measured via TGA experiment performed on a completely infiltrated HACNT–polystyrene composite using a TA Q500 (air, ramp rate 10.00 °C/min to 900.00 °C). Examination of the microstructural alignment in the as-grown HACNTs film was carried out with SEM (JEOL6340F FEGSEM, acceleration voltage = 5 kV, working distance = 6 mm) and was supported by wide-angle XRD data acquired using a Bruker NanoStar (Cu Kα radiation and 2D Hi star detector). For TEM studies, a sample of the HACNT film was removed from the silica substrates and dispersed onto holey carbon copper grids. The TEM micrographs were taken using a JEOL JEM-4000EX II (400 kV) microscope.

All the reagents (purity grade 99+%) were purchased from Acros Organics and were used without any further purification.
